# Analysis of Price Dynamic Competition and Stability in Cross-Border E-Commerce Supply Chain Channels Empowered by Blockchain Technology

**DOI:** 10.3390/e27101076

**Published:** 2025-10-16

**Authors:** Le-Bin Wang, Jian Chai, Lu-Ying Wen

**Affiliations:** 1 School of Economics and Management, Xidian University, Xi’an 710126, China; 2 School of Management, Xi’an Jiaotong University, Xi’an 710049, China

**Keywords:** blockchain, cross-border e-commerce, sales mode, price competition, stability, entropy, 91A25

## Abstract

Based on the perspective of multi-stage dynamic competition, this study constructs a discrete dynamic model of price competition between the “direct sales” and “resale” channels in cross-border e-commerce (CBEC) under three blockchain deployment modes. Drawing on nonlinear dynamics theory, the Nash equilibrium of the system and its stability conditions are examined. Using numerical simulations, the effects of factors such as the channel price adjustment speed, tariff rate, and commission ratio on the dynamic evolution, entropy, and stability of the system under the empowerment of blockchain technology are investigated. Furthermore, the impact of noise factors on system stability and the corresponding chaos control strategies are further analyzed. This study finds that a single-channel deployment tends to induce asymmetric system responses, whereas dual-channel collaborative deployment helps enhance strategic coordination. An increase in price adjustment speed, tariffs, and commission rates can drive the system’s pricing dynamics from a stable state into chaos, thereby raising its entropy, while the adoption of blockchain technology tends to weaken dynamic stability. Therefore, after deploying blockchain technology, each channel should make its pricing decisions more cautiously. Moderate noise can exert a stabilizing effect, whereas excessive disturbances may cause the system to diverge. Hence, enterprises should carefully assess the magnitude of disturbances and capitalize on the positive effects brought about by moderate fluctuations. In addition, the delayed feedback control method can effectively suppress chaotic fluctuations and enhance system stability, demonstrating strong adaptability across different blockchain deployment modes.

## 1. Introduction

With the continuous development of information technology and digital infrastructure, the importance of cross-border e-commerce (CBEC) platforms in global trade continues to rise. E-commerce platforms not only perform retail functions, but also act as product resellers, forming a channel structure in which “direct sales” and “resale” models coexist and compete intensively. According to data released by JD.com, in 2018, the revenues of self-operated (direct sales) channels and third-party (resale) sellers reached 461.1 billion yuan and CNY 45.9 billion, respectively [1]. At the same time, blockchain technology, with its transparency, traceability, and tamper-proof characteristics, has demonstrated unique value in addressing problems such as information asymmetry and product traceability difficulties in cross-border trade scenarios [2,3]. In recent years, many CBEC platforms have sought to introduce blockchain technology. For example, Alibaba’s Tmall Global and Ant Blockchain have jointly launched the “Product Traceability Code” initiative to enhance the anti-counterfeiting capabilities and traceability of imported goods.

Although the deployment of blockchain systems can provide tangible technical benefits, it also inevitably entails significant cost burdens. Supply chain entities hold differing views on whether to adopt blockchain technology and how to deploy it across different sales channels, thereby increasing the complexity of system coordination and decision-making. Therefore, studying the dynamics of price competition and its evolutionary mechanisms among CBEC sales channels under the empowerment of blockchain technology is not only of theoretical significance for understanding strategic interactions within the current digital supply chain environment, but also offers important decision-making support for enterprises aiming to design efficient and coordinated technology deployment and pricing strategies. The rapid development of e-commerce platforms has drawn increasing attention from the academic community to their operational and managerial issues. In particular, how to coordinate benefit distribution and strategic interactions in the e-commerce supply chain under the “resale” and “direct sales” models has become a research hotspot. On the one hand, some scholars have focused on the influencing factors of the two sales models and the optimal decisions of supply chain entities. Abhishek et al. [4] examined sales model selection for e-commerce platforms under the influence of externalities by developing a price competition model. The results indicated that the platform prefers the flagship store model when flagship store sales generate negative externalities for traditional channels, and prefers self-operated sales when self-operated sales generate positive externalities. Wei et al. [5] found that, under two distinct power structures, suppliers adopting the self-operated model represent the optimal decision for e-commerce platforms. Zennyo et al. [6] analyzed the sales model selection problem of two types of suppliers within a monopoly platform. Liu et al. [7] considered market size to explore how platforms choose between the resale (self-operated) and agency (third-party merchant) models. Zhang et al. [8] investigated the strategic trade-offs in manufacturers’ sales model selection and its impact on profits and environmental sustainability in the context of e-tailers introducing private brands, highlighting the key roles of commission ratio, brand advantage, and cost structure. On the other hand, some scholars have examined the coordination between sales model selection and other supply chain decisions. Zhang [9] incorporated information-sharing behavior into the analysis of sales model choice and found that e-commerce platforms are more willing to share information under the flagship store model while retaining information under the self-operated model. Yang et al. [10] explored the interaction between e-commerce information-sharing behavior and sales model selection and found that the platform’s strategy depends on the investment efficiency level of manufacturers. Based on a dual-channel supply chain framework, Yang et al. [11] examined the green investment strategy of e-commerce firms under carbon emission reduction policies and its interaction with manufacturers’ sales model choices. Wang et al. [12] analyzed the impact of different sales models (resale and direct sales) on the optimal trade-in strategies of suppliers in an e-commerce environment and found that the choice of sales model significantly affects both parties’ decisions. Although the above studies have investigated the strategic selection of sales models in e-commerce supply chains from multiple perspectives, they largely overlook the impact of blockchain technology on supply chain decision-making within the context of digitalization.

With its characteristics of decentralization, immutability, and traceability, blockchain technology is accelerating the transformation of supply chain management and has seen widespread application in recent years [13,14,15]. Existing studies on blockchain mainly focus on the operational management and contract coordination of single-channel supply chain entities [16,17], while other studies examine the coordination and decision-making issues within dual-channel supply chains that involve e-commerce platforms [18]. Xu et al. [19] investigated the linkage between sales model selection and blockchain adoption in manufacturers’ online–offline dual-channel sales under a cap-and-trade system and examined the impact of cross-channel effects, platform power, and commission rates on the profits and decisions of supply chain entities. Xia et al. [20] developed a dual-channel green supply chain model comprising manufacturers and e-commerce platforms and analyzed manufacturers’ optimal blockchain adoption decisions under different financing strategies. Lu et al. [21] explored the impact of different blockchain adoption models on consumer trust and channel performance in dual-channel supply chains. Zhao et al. [22] examined manufacturers’ strategic decisions to adopt blockchain technology within supply chains operating under the resale and direct sales models and found that these decisions were influenced by blockchain costs and commission rates and that blockchain adoption does not necessarily lead to increased demand. A comprehensive review of the above studies on dual-channel supply chains [18,19,20,21,22] reveals that researchers have conducted in-depth discussions of the strategic decision-making behaviors of manufacturers and e-commerce platforms under the empowerment of blockchain technology, but have yet to explore its impact and role from the perspective of cross-border trade.

At present, CBEC platforms have become a major engine driving the growth of global cross-border trade [23]. According to the latest data from eMarketer and Statista, the global CBEC transaction volume is projected to reach USD 2.1 trillion in 2024, representing an increase of about 18% compared with the previous year [24]. The application of blockchain technology in the field of cross-border trade has also made significant progress. Many large multinational companies and financial institutions have jointly launched blockchain-based cross-border trade platforms aimed at simplifying trade processes and improving transaction efficiency, such as the Conflux Network platform [25]. In this context, several scholars have examined the application and effectiveness of blockchain technology in CBEC and its impact on supply chain management and operational performance using empirical approaches. Lee et al. [26] conducted a pilot test of a blockchain-based cross-border traceability system, providing empirical evidence that blockchain—when integrated with complementary technologies—can enhance supply chain transparency, strengthen end-customer trust, and reduce the circulation of counterfeit goods. Jiang [27] performed a theoretical and empirical analysis of blockchain-based business models for cross-border e-commerce platforms, integrating case study results to explore the feasibility and application potential of blockchain in promoting cross-border trade and business model innovation. Tian et al. [25] carried out a field experiment on a blockchain-based platform facilitating cross-border trade between Yunnan Province, China, and Southeast Asian countries, and found that blockchain implementation significantly reduced operational, agency, and time costs, improved payment settlement efficiency, and enhanced interdepartmental business collaboration.

Furthermore, some scholars have explored operational management issues in the context of CBEC by developing methods or constructing models. Liu et al. [28] proposed a set of methods and frameworks based on blockchain technology to achieve product and transaction traceability within cross-border electronic supply chain management. Zhou et al. [29] developed an evolutionary game model between CBEC platforms and merchants to analyze the adoption of blockchain technology, revealing that the adoption likelihood depends on profit distribution and information costs, with both parties gradually converging toward blockchain adoption as the game evolves. Jiang et al. [30] analyzed the impact of blockchain adoption in cross-border settings on the operational decisions and profits of manufacturers and CBEC platforms, considering factors such as tax differences, market competition, and consumer preferences, within a cross-border dual-channel supply chain model. Although these studies explored the decision-making interaction between manufacturers and e-commerce platforms under blockchain technology in cross-border contexts, they did not investigate the complexity and multi-stage dynamic evolution patterns of such decision-making behaviors under blockchain empowerment [31]. In actual operations, supply chain companies continuously adjust the pricing of different product lines as consumer demand changes and blockchain costs fluctuate. For example, Louis Vuitton, a subsidiary of the LVMH Group, launched the Aura blockchain platform to track the entire production process, thereby improving traceability, transparency, and anti-counterfeiting capabilities. The company also implemented differentiated pricing across different markets for certain limited editions through this system to cover costs and enhance perceived brand value. Similarly, in response to the European and American markets’ emphasis on traceability, the global tea brand Teabox introduced a blockchain traceability system for selected product lines to enhance consumer trust and flexibly adjust pricing strategies based on changes in system construction and maintenance costs [32]. However, frequent adjustments of prices or other operational decisions may induce nonlinear dynamic effects and potential instabilities in supply chain systems [33]. Consequently, entropy-based methods have been widely employed to analyze the complexity and uncertainty in supply chain decision-making. For example, Petridis et al. [34] employed an entropy-based approach to investigate the impact of stochastic fluctuations in production and transportation costs on supply chain decision-making, demonstrating how uncertainty can be incorporated into cost and pricing optimization. Xie et al. [23] combined game theory with complex systems theory and employed discrete dynamic models and entropy analysis to investigate the impact of different logistics modes and information-sharing strategies on output decision stability in closed-loop cross-border e-commerce supply chains, finding that controlling initial output levels and tariff rates can effectively suppress system chaos and reduce entropy, thereby restoring decision stability. Mishra et al. [35] applied an entropy-based approach to evaluate blockchain platforms for healthcare supply chains, emphasizing the role of entropy in capturing uncertainty within multi-criteria decision-making.

While entropy-based methods effectively capture the inherent complexity and uncertainty of supply chain decision-making, the multi-stage nature of cross-border supply chains introduces additional sources of variability, particularly from random disturbances such as exchange rate fluctuations, price shocks, and trade policy changes. With the deepening of related research, scholars have gradually recognized the complex impact mechanisms of such random disturbances on market dynamics and have explored their influence on system behavior and stability characteristics from multiple perspectives [36,37]. For example, several studies have revealed that noise can, in some cases, enhance system stability—a phenomenon known as the “noise-enhanced stability” or “noise enhancement effect”, as observed in metastable states [38,39]. In addition, although the introduction of blockchain has strengthened information-sharing mechanisms, differences in on-chain data availability and analytical capabilities may lead to strategic misjudgments during actual game processes, thereby exacerbating uncertainty in cross-border supply chain decision-making and posing challenges to system stability [40]. Therefore, traditional deterministic analytical methods can no longer fully capture the actual dynamic characteristics of CBEC supply chain systems empowered by blockchain technology. To construct a more explanatory and realistic dynamic model, it is essential to introduce a noise-driven mechanism and integrate it with nonlinear systems theory to more accurately characterize system behavior.

Motivated by the above discussion, this study develops a dynamic model for pricing decision-making in the two sales modes (“direct sales” and “resale”) of CBEC platforms, explicitly capturing the multi-stage strategic interactions between manufacturers and platforms, thereby addressing the existing gap in multi-stage dynamic analysis. By incorporating nonlinear dynamic theory and noise-driven mechanisms, the model further investigates how channel price adjustment speed, tax rates, commission ratios, and stochastic disturbances influence the evolutionary behavior and stability of the system, thereby addressing the limited scholarly attention to stochastic drivers and nonlinear effects within cross-border supply chains. Finally, using numerical simulations, this study derives actionable managerial implications for platform operators and channel members, providing practical guidance for strategic formulation in complex and uncertain market environments and bridging the gap between theoretical modeling and real-world decision-making.

The remainder of this paper is organized as follows: Section 2 introduces the research problem and formulates the model. Section 3 derives the Nash equilibrium solutions and analyzes their stability conditions. Section 4 employs numerical simulation methods to investigate the system’s dynamic evolution, examining the effects of noise and other factors on the system’s entropy and stability, as well as proposing corresponding chaos control strategies. Finally, Section 5 concludes the paper and summarizes the main findings.

## 2. Problem Description and Model Building

**Figure 1:**
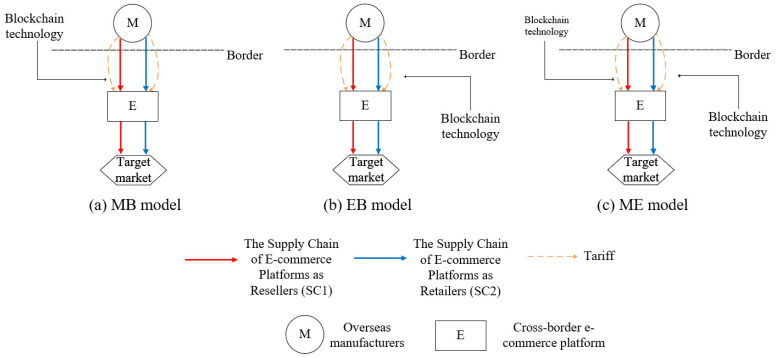
Cross-border e-commerce supply chain system for product P*.

This study focuses on the CBEC supply chain system comprising overseas manufacturers (denoted as M) and CBEC platforms (denoted as E). In this system, there are two sales channels for product P*: sales channel 1 (denoted as SC1) where E acts as a reseller and sales channel 2 (denoted as SC2), where E functions as a retailer. In SC1, manufacturer M puts product P* on the platform E, and manufacturer M sets the price independently and pays a certain percentage of commission to platform E. Platform E serves as a channel intermediary, primarily responsible for product display and transaction matchmaking, but does not participate in pricing decisions. In this channel, the pricing power of the supply chain is characterized by manufacturer dominance, such as the sales channel composed of red arrows in Figure 1. In SC2, M sells product P* to platform E at wholesale prices, and the platform sets the retail price and sells it to end consumers. Therefore, in this channel, the platform assumes the functions of pricing and sales, forming a supply chain structure dominated by platform pricing, such as the sales channel composed of blue arrows in Figure 1. In the above supply chain system, blockchain technology can be deployed in different modes. This paper considers the following three modes: MB mode (M-led construction): blockchain is deployed only in channel SC1, and the platform does not participate; EB mode (E-led construction): blockchain is deployed only in channel SC2, and manufacturers do not participate; ME mode (M and E co-construction): SC1 and SC2 both introduce blockchain, and the cost and maintenance responsibilities shared by both parties. In addition, the yellow dotted arrow shown in the figure represents the tariffs applied to P* in cross-border trade. It is worth noting that we use the term CBEC rather than global e-commerce because this study specifically examines trade between two countries or regions, rather than worldwide transactions.

The variables and symbols used in the model are shown in Table 1.

### 2.1. Demand Function

Consumers who purchase product P* through a CBEC platform (E) are required to pay the corresponding tariffs. Let r represent the tariff rate, then the actual price paid by the consumer is $p_{i}(1+r)$ ($i\in \left\{1,2\right\}$). On SC1, P* is priced by M. In this scenario, E is the reseller of product P* and charges a certain percentage of commission m to obtain revenue. Since M directly sets the price, this part of the commission cost will also be passed on to the end consumer, so the final sales price of P* on SC1 can be expressed as $p_{i}(1+r+m)$. In the MB mode, SC1 adopts blockchain technology, $b_{1}$ represents the maintenance effort of blockchain, and $g_{1}(g_{1}>0)$ represents the sensitivity coefficient of price with respect to the level of maintenance effort; a larger $g_{1}$ means that consumers are more sensitive to the level of blockchain technology. Therefore, similar to the approaches used by Zha et al. [24] and Ha et al. [41], the demand function of P* in the two supply chains in the MB mode is expressed as

(1) $$\begin{array}{l} q_{1}^{\mathrm{MB}}=a-p_{1}^{\mathrm{MB}}(1+r+m)+\beta p_{2}^{\mathrm{MB}}(1+r)+g_{1}b_{1}^{\mathrm{MB}}\\ q_{2}^{\mathrm{MB}}=a-p_{2}^{\mathrm{MB}}(1+r)+\beta p_{1}^{\mathrm{MB}}(1+r+m) \end{array}$$

where a denotes the initial market size of P*, and β∈(0,1) represents the competition intensity between the two channels.

Similarly, in the EB mode, SC2 deploys blockchain, $b_{2}$ represents the maintenance effort of the blockchain, and $g_{2}$ represents the sensitivity coefficient of price to the maintenance effort level. In the EB mode, the demand function of P* in the two supply chains is expressed as

(2) $$\begin{array}{l} q_{1}^{\mathrm{EB}}=a-p_{1}^{\mathrm{EB}}(1+r+m)+\beta p_{2}^{\mathrm{EB}}(1+r)\\ q_{2}^{\mathrm{EB}}=a-p_{2}^{\mathrm{EB}}(1+r)+\beta p_{1}^{\mathrm{EB}}(1+r+m)+g_{2}b_{2}^{\mathrm{EB}} \end{array}$$

In ME mode, SC1 and SC2 both deploy blockchain, then the demand functions of P* in the two supply chains can also be expressed as

(3) $$\begin{array}{l} q_{1}^{\mathrm{ME}}=a-p_{1}^{\mathrm{ME}}(1+r+m)+\beta p_{2}^{\mathrm{ME}}(1+r)+g_{1}b_{1}^{\mathrm{ME}}\\ q_{2}^{\mathrm{ME}}=a-p_{2}^{\mathrm{ME}}(1+r)+\beta p_{1}^{\mathrm{ME}}(1+r+m)+g_{2}b_{2}^{\mathrm{ME}} \end{array}$$

### 2.2. Profit Function

When SCi adopts blockchain technology, in addition to the fixed construction cost $A_{m}$, it will also incur blockchain maintenance costs. $\eta_{i}$ represents the maintenance cost coefficient. Similarly to the studies of Zha et al. [24] and Lou et al. [42], the blockchain maintenance cost that SCi needs to pay is $\frac{1}{2}\eta_{i}{\left(b_{i}\right)}^{2}$. In the MB mode, SCi’s profit is

(4) $$\begin{array}{l} \pi_{1}^{\mathrm{MB}}=(p_{1}^{\mathrm{MB}}(1+r+m)-c_{1})q_{1}^{\mathrm{MB}}-\eta_{1}^{\mathrm{MB}}{\left(b_{1}^{\mathrm{MB}}\right)}^{2}/2-A_{m}\\ \pi_{2}^{\mathrm{MB}}=(p_{2}^{\mathrm{MB}}(1+r)-c_{2})q_{2}^{\mathrm{MB}} \end{array}$$

where $c_{i}$ represents the unit production cost of SCi for product P*.

In the EB mode, the profits of the two supply chains SCi are

(5) $$\begin{array}{l} \pi_{1}^{\mathrm{EB}}=(p_{1}^{\mathrm{EB}}(1+r+m)-c_{1})q_{1}^{\mathrm{EB}}\\ \pi_{2}^{\mathrm{EB}}=(p_{2}^{\mathrm{EB}}(1+r)-c_{2})q_{2}^{\mathrm{EB}}-\eta_{2}^{\mathrm{EB}}{\left(b_{2}^{\mathrm{EB}}\right)}^{2}/2-A_{m} \end{array}$$

In the ME mode, the two channels SCi jointly bear the blockchain construction cost $A_{m}$ and jointly invest in maintaining the blockchain. Let $\varepsilon_{1}$ be the blockchain cost sharing coefficient of SC1, and $1-\varepsilon_{1}$ be the cost sharing coefficient of SC2. Then the profits of the two channels SCi are

(6) $$\begin{array}{l} \pi_{1}^{\mathrm{ME}}=(p_{1}^{\mathrm{ME}}(1+r+m)-c_{1})q_{1}^{\mathrm{ME}}-\eta_{1}^{\mathrm{ME}}{\left(b_{1}^{\mathrm{ME}}\right)}^{2}/2-\varepsilon_{1}A_{m}\\ \pi_{2}^{\mathrm{ME}}=(p_{2}^{\mathrm{ME}}(1+r)-c_{2})q_{2}^{\mathrm{ME}}-\eta_{2}^{\mathrm{ME}}{\left(b_{2}^{\mathrm{ME}}\right)}^{2}/2-(1-\varepsilon_{1})A_{m} \end{array}$$

### 2.3. Discrete Dynamics Model Construction

For expression (4), the first-order partial derivatives of $\pi_{i}^{\mathrm{MB}}$ with respect to $p_{i}^{\mathrm{MB}}$ and $b_{i}^{\mathrm{MB}}$ in MB mode can be calculated as shown in expression (7).

(7) $$\begin{array}{l} \frac{\partial \pi_{1}^{\mathrm{MB}}}{\partial p_{1}^{\mathrm{MB}}}=(1+r+m)(q_{1}^{\mathrm{MB}}-p_{1}^{\mathrm{MB}}(1+r+m)+c_{1})\\ \frac{\partial \pi_{1}^{\mathrm{MB}}}{\partial b_{1}^{\mathrm{MB}}}=(p_{1}^{\mathrm{MB}}(1+r+m)-c_{1})g_{1}^{\mathrm{MB}}-\eta_{1}^{\mathrm{MB}}b_{1}^{\mathrm{MB}}\\ \frac{\partial \pi_{2}^{\mathrm{MB}}}{\partial p_{2}^{\mathrm{MB}}}=(1+r)(q_{2}^{\mathrm{MB}}-p_{2}^{\mathrm{MB}}(1+r)+c_{2}) \end{array}$$

For expression (5), the first-order partial derivatives of $\pi_{i}^{\mathrm{EB}}$ with respect to $p_{i}^{\mathrm{EB}}$ and $b_{i}^{\mathrm{MB}}$ in ME mode can be calculated as shown in expression (8).

(8) $$\begin{array}{l} \frac{\partial \pi_{1}^{\mathrm{EB}}}{\partial p_{1}^{\mathrm{EB}}}=(1+r+m)(q_{1}^{\mathrm{EB}}-p_{1}^{\mathrm{EB}}(1+r+m)+c_{1})\\ \frac{\partial \pi_{2}^{\mathrm{EB}}}{\partial p_{2}^{\mathrm{EB}}}=(1+r)(q_{2}^{\mathrm{EB}}-p_{2}^{\mathrm{EB}}(1+r)+c_{2})\\ \frac{\partial \pi_{2}^{\mathrm{EB}}}{\partial b_{2}^{\mathrm{EB}}}=(p_{2}^{\mathrm{EB}}(1+r)-c_{2})g_{2}^{\mathrm{EB}}-\eta_{2}^{\mathrm{EB}}b_{2}^{\mathrm{EB}} \end{array}$$

For expression (6), the first-order partial derivatives of $\pi_{i}^{\mathrm{ME}}$ with respect to $p_{i}^{\mathrm{ME}}$ and $b_{i}^{\mathrm{ME}}$ in ME modes can be calculated as shown in expression (9).

(9) $$\begin{array}{l} \frac{\partial \pi_{1}^{\mathrm{ME}}}{\partial p_{1}^{\mathrm{ME}}}=(1+r+m)(q_{1}^{\mathrm{ME}}-p_{1}^{\mathrm{ME}}(1+r+m)+c_{1})\\ \frac{\partial \pi_{2}^{\mathrm{ME}}}{\partial p_{2}^{\mathrm{ME}}}=(1+r)(q_{2}^{\mathrm{ME}}-p_{2}^{\mathrm{ME}}(1+r)+c_{2})\\ \frac{\partial \pi_{1}^{\mathrm{ME}}}{\partial b_{1}^{\mathrm{ME}}}=(p_{1}^{\mathrm{ME}}(1+r+m)-c_{1})g_{1}^{\mathrm{ME}}-\eta_{1}^{\mathrm{ME}}b_{1}^{\mathrm{ME}}\\ \frac{\partial \pi_{2}^{\mathrm{ME}}}{\partial b_{2}^{\mathrm{ME}}}=(p_{2}^{\mathrm{ME}}(1+r)-c_{2})g_{2}^{\mathrm{ME}}-\eta_{2}^{\mathrm{ME}}b_{2}^{\mathrm{ME}} \end{array}$$

Supply chain participants (M and E) often do not have complete information and cannot perfectly or accurately predict future market changes. They cannot directly calculate the optimal pricing decision for profit maximization, but instead continuously adjust their price decisions based on changes in marginal profits to maximize returns. This decision-making approach, based on marginal profit adjustment, is referred to as bounded rational decision-making. In addition, since the blockchain maintenance efforts of SCi at each stage will incur certain costs, which in turn affect profits and ultimately affect product pricing, the price decision-making system studied in this paper also considers the impact of blockchain maintenance effort decisions. Similarly to Lou et al. [42] and Ma et al. [43], it is assumed that the adjustment of SCi’s price and blockchain maintenance effort decision in period t+1 is proportional to the marginal profit in period t, that is $\frac{p_{i,t+1}^{\delta }-p_{i,t}^{\delta }}{p_{i,t}^{\delta }}=\omega_{i}^{\delta }\frac{\partial \pi_{i,t}^{\delta }}{\partial p_{i,t}^{\delta }}$, $\frac{b_{i,t+1}^{\delta }-b_{i,t}^{\delta }}{b_{i,t}^{\delta }}=\mu_{i}^{\delta }\frac{\partial \pi_{i,t}^{\mathrm{MB}}}{\partial b_{i,t}^{\mathrm{MB}}}$, $i\in \left\{1,2\right\},\delta \in \left\{\mathrm{MB},\mathrm{EB},\mathrm{ME}\right\}$, where $\omega_{i}^{\delta }$ represents the adjustment speed of SCi’s price decision and $\mu_{i}^{\delta }$ represents the adjustment speed of SCi’s blockchain maintenance effort decision.

Then, in the MB mode, the discrete dynamics model of SCi price decision can be expressed as

(10) $$\begin{array}{l} p_{i,t+1}^{\mathrm{MB}}=p_{i,t}^{\mathrm{MB}}+\omega_{i}^{\mathrm{MB}}p_{i,t}^{\mathrm{MB}}\frac{\partial \pi_{i,t}^{\mathrm{MB}}}{\partial p_{i,t}^{\mathrm{MB}}},i=1,2\\ b_{1,t+1}^{\mathrm{MB}}=b_{1,t}^{\mathrm{MB}}+\mu_{1}^{\mathrm{MB}}b_{1,t}^{\mathrm{MB}}\frac{\partial \pi_{1,t}^{\mathrm{MB}}}{\partial b_{1,t}^{\mathrm{MB}}} \end{array}$$

In the EB mode, the discrete dynamics model of SCi price decision can be expressed as

(11) $$\begin{array}{l} p_{i,t+1}^{\mathrm{EB}}=p_{i,t}^{\mathrm{EB}}+\omega_{i}^{\mathrm{EB}}p_{i,t}^{\mathrm{EB}}\frac{\partial \pi_{i,t}^{\mathrm{EB}}}{\partial p_{i,t}^{\mathrm{EB}}},i=1,2\\ b_{2,t+1}^{\mathrm{EB}}=b_{2,t}^{\mathrm{EB}}+\mu_{2}^{\mathrm{EB}}b_{2,t}^{\mathrm{EB}}\frac{\partial \pi_{2,t}^{\mathrm{EB}}}{\partial b_{2,t}^{\mathrm{EB}}} \end{array}$$

In the ME mode, the discrete dynamics model of SCi price decision can be expressed as

(12) $$\begin{array}{l} p_{i,t+1}^{\mathrm{ME}}=p_{i,t}^{\mathrm{ME}}+\omega_{i}^{\mathrm{ME}}p_{i,t}^{\mathrm{ME}}\frac{\partial \pi_{i,t}^{\mathrm{ME}}}{\partial p_{i,t}^{\mathrm{ME}}},i=1,2\\ b_{i,t+1}^{\mathrm{ME}}=b_{i,t}^{\mathrm{ME}}+\mu_{i}^{\mathrm{ME}}b_{i,t}^{\mathrm{ME}}\frac{\partial \pi_{i,t}^{\mathrm{ME}}}{\partial b_{i,t}^{\mathrm{ME}}},i=1,2 \end{array}$$

## 3. Nash Equilibrium Solution and Stability Conditions

Let $p_{i,t+1}^{\delta }=p_{i,t}^{\delta }$ and $b_{i,t+1}^{\delta }=b_{i,t}^{\delta }$ in the discrete dynamics model (10), (11) and (12), respectively, we can obtain 8 equilibrium solutions of the model in MB and EB mode, and 16 equilibrium solutions of the model in ME mode. Where MB, EB and ME modes each have only one equilibrium solution $E^{\mathrm{MB}}=(p_{1}^{{\mathrm{MB}}^{*}},p_{2}^{{\mathrm{MB}}^{*}},b_{1}^{{\mathrm{MB}}^{*}})$, $E^{\mathrm{EB}}=(p_{1}^{{\mathrm{EB}}^{*}},p_{2}^{{\mathrm{EB}}^{*}},b_{2}^{{\mathrm{EB}}^{*}})$, and there is no decision with a value of 0. At this time, the two SCi cannot act independently to increase profits. Therefore, $E^{\mathrm{MB}}$, $E^{\mathrm{EB}}$ and $E^{\mathrm{ME}}$ are the Nash equilibrium points of the system in MB, EB and ME modes, respectively, and their specific expressions are shown in Appendix A.

To investigate the local stability of the Nash equilibrium points of the discrete dynamic model defined by Equations (10)–(12), it is first necessary to compute the system’s Jacobian matrix. Since the discrete dynamic models in the MB and EB modes are three-dimensional iterative systems, the Jacobian matrices for the models in the MB and EB modes are derived as follows:

(13) $$J^{\delta }=\left(\begin{array}{ccc} j_{11}^{\delta } & j_{12}^{\delta } & j_{13}^{\delta }\\ j_{21}^{\delta } & j_{22}^{\delta } & j_{23}^{\delta }\\ j_{31}^{\delta } & j_{32}^{\delta } & j_{33}^{\delta } \end{array}\right),\delta \in \left\{\mathrm{MB},\mathrm{EB}\right\}$$

where the specific expression of $J^{\delta },\delta \in \left\{\mathrm{MB},\mathrm{EB}\right\}$ is shown in Appendix B.

The characteristic polynomial of the Jacobian matrix (13) is

(14) $$f^{\delta }(\lambda )=\lambda^{3}+B_{1}^{\delta }\lambda^{2}+B_{2}^{\delta }\lambda +B_{3}^{\delta },\delta \in \left\{\mathrm{MB},\mathrm{EB}\right\}$$

where

$B_{1}^{\delta }=-j_{11}^{\delta }-j_{22}^{\delta }-j_{33}^{\delta }$.

$B_{2}^{\delta }=-j_{12}^{\delta }j_{21}^{\delta }+j_{11}^{\delta }j_{22}^{\delta }-j_{13}^{\delta }j_{31}^{\delta }-j_{23}^{\delta }j_{32}^{\delta }+j_{11}^{\delta }j_{33}^{\delta }+j_{22}^{\delta }j_{33}^{\delta }$.

$B_{3}^{\delta }=j_{13}^{\delta }j_{22}^{\delta }j_{31}^{\delta }-j_{12}^{\delta }j_{23}^{\delta }j_{31}^{\delta }-j_{13}^{\delta }j_{21}^{\delta }j_{32}^{\delta }+j_{11}^{\delta }j_{23}^{\delta }j_{32}^{\delta }+j_{12}^{\delta }j_{21}^{\delta }j_{33}^{\delta }-j_{11}^{\delta }j_{22}^{\delta }j_{33}^{\delta }$.

By substituting the values of each equilibrium point into the Jacobian matrix, the corresponding eigenvalues can be obtained. The local stability of each equilibrium point can then be determined based on whether the magnitudes of its eigenvalues are less than 1. According to the Jury stability criterion [44], if the conditions specified in expression (15) are satisfied, all the eigenvalues of the discrete dynamic system lie within the unit circle. Consequently, the corresponding equilibrium point is locally asymptotically stable.

(15) $$\left\{\begin{array}{l} f^{\sigma }(1)=1+B_{1}^{\sigma }+B_{2}^{\sigma }+B_{3}^{\sigma }>0\\ -f^{\sigma }(-1)=1-B_{1}^{\sigma }+B_{2}^{\sigma }-B_{3}^{\sigma }>0\\ \left|B_{3}^{\sigma }\right|<1\\ \left|B_{3}^{\sigma^{2}}-1\right|>\left|B_{1}^{\sigma }B_{3}^{\sigma }-B_{2}^{\sigma }\right| \end{array}\right.,\delta \in \left\{\mathrm{MB},\mathrm{EB}\right\}$$

Thus, the region of $\omega_{i}^{\delta }$, and $\mu_{i}^{\delta }$ formed by expression (15) is the stable domain of systems (10) and (11). When the values of $\omega_{i}^{\delta }$ and $\mu_{i}^{\delta }$ are within this stable domain, the price of SCi and the blockchain effort level decision will gradually stabilize to the Nash equilibrium point $E^{\delta }$, $\delta \in \left\{\mathrm{MB},\mathrm{EB}\right\}$ in the competition.

The discrete dynamics model in the ME mode is a four-dimensional iterative system, so the Jacobian matrix of the model in the ME mode can be obtained as

(16) $$J^{\mathrm{ME}}=\left(\begin{array}{cccc} j_{11}^{\mathrm{ME}} & j_{12}^{\mathrm{ME}} & j_{13}^{\mathrm{ME}} & j_{14}^{\mathrm{ME}}\\ j_{21}^{\mathrm{ME}} & j_{22}^{\mathrm{ME}} & j_{23}^{\mathrm{ME}} & j_{24}^{\mathrm{ME}}\\ j_{31}^{\mathrm{ME}} & j_{32}^{\mathrm{ME}} & j_{33}^{\mathrm{ME}} & j_{34}^{\mathrm{ME}}\\ j_{41}^{\mathrm{ME}} & j_{42}^{\mathrm{ME}} & j_{43}^{\mathrm{ME}} & j_{44}^{\mathrm{ME}} \end{array}\right)$$

where the specific expression of $J^{\mathrm{ME}}$ is shown in Appendix C.

The characteristic polynomial of the Jacobian matrix (16) is

(17) $$f^{\mathrm{ME}}(\lambda )=\lambda^{4}+B_{1}^{\mathrm{ME}}\lambda^{3}+B_{2}^{\mathrm{ME}}\lambda^{2}+B_{3}^{\mathrm{ME}}\lambda +B_{4}^{\mathrm{ME}}$$

where the specific expression of $J^{\mathrm{ME}}$ is shown in Appendix D.

According to the Jury criterion, the condition that the discrete dynamic system (12) needs to satisfy for asymptotic stability is

(18) $$\left\{\begin{array}{l} f^{\mathrm{ME}}(1)=1+B_{1}^{\mathrm{ME}}+B_{2}^{\mathrm{ME}}+B_{3}^{\mathrm{ME}}+B_{4}^{\mathrm{ME}}>0\\ f^{\mathrm{ME}}(-1)=1-B_{1}^{\sigma }+B_{2}^{\sigma }-B_{3}^{\sigma }+B_{4}^{\mathrm{ME}}>0\\ \begin{array}{l} \left|B_{4}^{\mathrm{ME}}\right|<1\\ \left|\theta_{0}\right|<\left|\theta_{3}\right| \end{array}\\ \left|\gamma_{0}\right|<\left|\gamma_{2}\right| \end{array}\right.$$

where $\theta_{3}=1-B_{4}^{{\mathrm{ME}}^{2}}$, $\theta_{2}=B_{1}^{\mathrm{ME}}-B_{3}^{\mathrm{ME}}B_{4}^{\mathrm{ME}}$, $\theta_{1}=B_{1}^{\mathrm{ME}}-B_{2}^{\mathrm{ME}}B_{4}^{\mathrm{ME}}$, $\theta_{0}=B_{3}^{\mathrm{ME}}-B_{1}^{\mathrm{ME}}B_{4}^{\mathrm{ME}}$, $\gamma_{2}=\theta_{3}^{2}-\theta_{0}^{2}$, $\gamma_{1}=\theta_{3}\theta_{2}-\theta_{1}\theta_{0}$, $\gamma_{0}=\theta_{3}\theta_{1}-\theta_{2}\theta_{0}$.

The region of $\omega_{i}^{\mathrm{ME}}$ and $\mu_{i}^{\mathrm{ME}}$ formed by expression (18) is called the stability domain of the discrete dynamic system (12). When the values of $\omega_{i}^{\mathrm{ME}}$ and $\mu_{i}^{\mathrm{ME}}$ are within this stability domain, the price and blockchain effort level decisions of SCi will gradually stabilize to the Nash equilibrium point $E^{\mathrm{ME}}$ in the competition.

## 4. Numerical Simulation Analysis

In view of the complexity of channel price competition in the CBEC supply chain studied in this paper, this section, based on the above theoretical analysis, uses numerical simulation methods to further explore the stability conditions and internal evolution laws of price competition decisions among channels enabled by blockchain technology, the impact of noise factors on system entropy and stability, and the chaos control methods of the system.

In this study, some parameters are directly adopted from the previous literature, while others are assumed for the purposes of model analysis. Specifically, the initial market size of P* is taken from Lou et al. [42], and the competition intensity between the two channels is adopted from Ma et al. [43], that is, β=1. Other parameters are assumed in this study to facilitate analysis: to examine the impact of different blockchain deployment modes on the stability of price competition between sales channels, we assume that consumers are more sensitive to the blockchain effort level $b_{i}^{\delta }$, that is, $g_{1}=g_{2}=1$. Considering that the construction of blockchain systems usually involves a one-time high fixed investment, we assume that the construction cost is $A_{m}=1$, while its subsequent maintenance costs are relatively low, and the maintenance cost coefficient is $\eta_{1}=\eta_{2}=0.1$. To facilitate the analysis of the impact of tariffs and commission rates on the dynamic evolution of the system, this paper sets the tariff rate to r=0.1 and the commission rate to m=0.1 in the baseline case. In the ME mode, M and E jointly invest in the construction of the blockchain, we assume that both parties share the construction costs equally, that is, $\varepsilon_{1}=0.5$. In addition, to ensure the non-negativity of profits, we assume that the unit production cost of product P* is lower, $c_{1}=c_{2}=0.1$.

### 4.1. Analysis of the Dynamic Evolution of System Price Decision-Making

**Figure 2:**
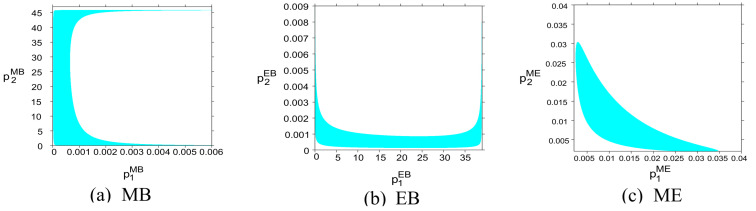
Two-dimensional stability domain of initial price decision $p_{i}^{\delta }$ in (**a**) MB, (**b**) EB, and (**c**) ME modes.

The adjustment speed of the fixed price and blockchain maintenance level is $\omega_{i}^{\delta }=u_{i}^{\delta }=0.01$. The stable domain formed by the initial price decisions of the two channels SCi in the three modes is shown in the cyan area in Figure 2a–c. When the initial price decision of SCi is within this area, after iteration, the price decision of the system will eventually converge to the Nash equilibrium point $E^{\delta }$, $\delta \in \left\{\mathrm{MB},\mathrm{EB},\mathrm{ME}\right\}$. Otherwise, the price decision of the system will eventually enter an unstable state.

As shown in Figure 2, in the MB mode (i.e., SC1 deploys blockchain technology), the stability range of SC1’s initial price decision does not exceed 0.006, while the stability range of SC2 is as high as 45, which is significantly larger than SC1. This shows that channels using blockchain technology are more sensitive to initial price decisions, and the tolerance range of price setting is significantly narrowed. Similarly, as shown in the figure, in the EB mode (i.e., SC2 deploys blockchain technology), the stability range of SC1’s initial price decision is also much larger than SC2, further confirming that blockchain technology will increase the sensitivity of channel price decisions. Therefore, when a channel adopts blockchain technology, CBEC supply chain enterprises should be more cautious when choosing the initial price strategy to ensure that they do not exceed the threshold shown in the figure to maintain the stability of the system. As shown in the figure, in the ME mode (i.e., both SC1 and SC2 deploy blockchain technology), the initial price decision stability range of the two channels is about 0.03, with a small difference. This shows that in the MB or EB mode, only one channel adopts blockchain technology, resulting in structural asymmetry in information processing and demand response between the two channels, making the initial price strategy of one party too sensitive and the other party slow to respond, which is easy to cause strategy imbalance and system instability. In the ME mode, the sensitivity of the two channels is symmetrical and the stability range is similar (about 0.03). Although the fault tolerance is reduced, the response consistency is enhanced, which helps to reduce asymmetric interference and improve the coordination and stability of the system. Therefore, when introducing blockchain technology, enterprises should not deploy it in isolation in only one channel or link, otherwise it may aggravate the asymmetry of information and strategy response. Therefore, from a managerial standpoint, when introducing blockchain technology, enterprises should avoid deploying it in isolation within a single channel or link. Instead, coordinated or synchronized adoption across key nodes of the supply chain can mitigate information asymmetry, enhance price coordination, and ensure long-term system stability. For CBEC practitioners, these findings suggest that blockchain investment decisions should be integrated into broader digital governance and collaborative pricing strategies, rather than being treated solely as technical upgrades. In this regard, blockchain’s value in CBEC lies not only in enhancing transparency and trust, but also in its capacity to reshape multi-agent coordination mechanisms in dynamic global markets.

**Figure 3:**
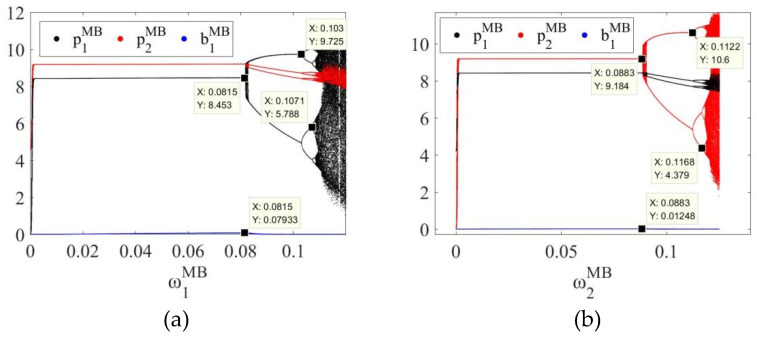
Bifurcation diagram of the system in the MB mode with respect to (**a**) the price adjustment speed $\omega_{1}^{\mathrm{MB}}$ of SC1 and (**b**) the price adjustment speed $\omega_{2}^{\mathrm{MB}}$ of SC2.

**Figure 4:**
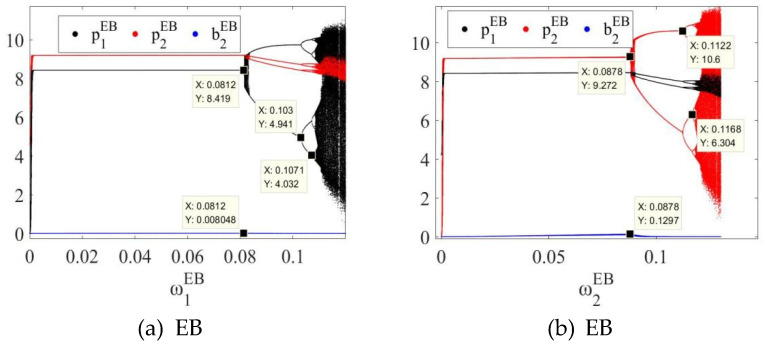
Bifurcation diagram of the system in the EB mode with respect to (**a**) the price adjustment speed $\omega_{1}^{\mathrm{EB}}$ of SC1 and (**b**) the price adjustment speed $\omega_{2}^{\mathrm{EB}}$ of SC2.

**Figure 5:**
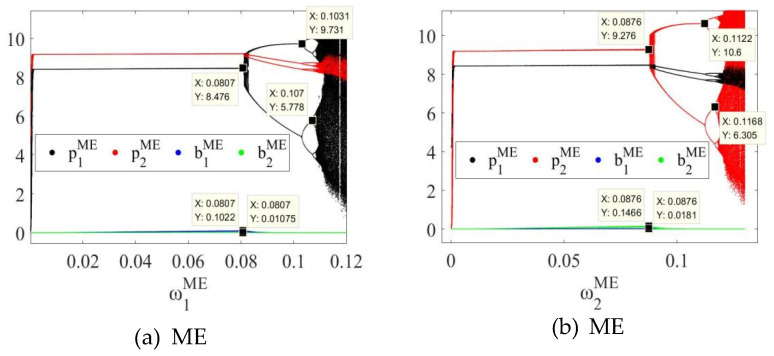
Bifurcation diagram of the system in the ME mode with respect to (**a**) the price adjustment speed $\omega_{1}^{\mathrm{ME}}$ of SC1 and (**b**) the price adjustment speed $\omega_{2}^{\mathrm{ME}}$ of SC2.

In order to further explore the dynamic evolution law of the system, by fixing $u_{i}^{\delta }=0.01$, the bifurcation diagrams of the system in MB, EB and ME modes as the price adjustment speed $\omega_{i}^{\delta }$ of SCi changes can be obtained, as shown in Figure 3a,b, Figure 4a,b and Figure 5a,b, respectively.

In the three modes, as the price adjustment speed $\omega_{i}^{\delta }$ increases, the system gradually transitions from a stable state to a period-doubling bifurcation state, and finally enters a chaotic state. For example, as shown in Figure 3, in the MB mode, when $0<\omega_{1}^{\mathrm{MB}}<0.0815$ or $0<\omega_{2}^{\mathrm{MB}}<0.0883$, the system is in a stable state; when $\omega_{1}^{\mathrm{MB}}=0.0815$ or $\omega_{2}^{\mathrm{MB}}=0.0883$, the system loses stability and has the first bifurcation; when $0.0815<\omega_{1}^{\mathrm{MB}}<0.1030$ or $0.0883<\omega_{2}^{\mathrm{MB}}<0.1122$, the system enters a two-period bifurcation state; when $\omega_{1}^{\mathrm{MB}}=0.1030$ or $\omega_{2}^{\mathrm{MB}}=0.1122$, the system has a second bifurcation; when $0.1030<\omega_{1}^{\mathrm{MB}}<0.1071$ or $0.1122<\omega_{2}^{\mathrm{MB}}<0.1168$, the system enters a four-period bifurcation state; as $\omega_{1}^{\mathrm{MB}}$ or $\omega_{2}^{\mathrm{MB}}$ further increases, the system eventually enters a chaotic state. In the EB and ME modes, the system also has similar dynamic evolution laws, as shown in Figure 4 and Figure 5. Therefore, regardless of whether blockchain technology is deployed, overseas manufacturers (M) and CBEC platforms (E) should control the price adjustment speed when formulating pricing strategies to maintain system stability. The specific stability thresholds are: the stability thresholds of the price adjustment speed of SC1 in the MB mode are 0.0815 and SC2 are 0.0883; SC1 in the EB mode is 0.0812 and SC2 is 0.0878; SC1 in the ME mode is 0.0807 and SC2 is 0.0876.

In addition, the comparison of Figure 3, Figure 4 and Figure 5 reveals that when both channels in the system use blockchain technology, the stability thresholds of SC1 and SC2 are reduced. This shows that although blockchain improves information transparency and response speed, it also enhances the system’s sensitivity to price adjustments and reduces the tolerance boundary of dynamic stability. Therefore, under the full deployment of blockchain, enterprises need to carefully control the rhythm of price adjustments to avoid causing system shocks.

**Figure 6:**
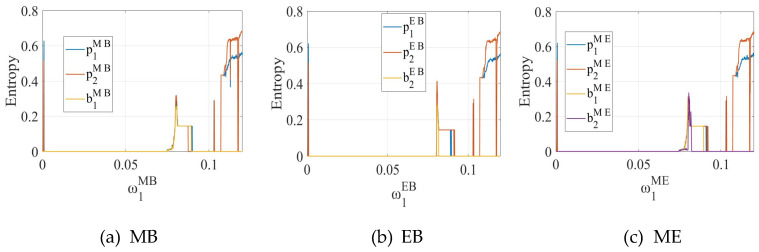
Entropy diagrams of the system with respect to the price adjustment speed $\omega_{1}^{\delta }$ of SC1 in the (**a**) MB, (**b**) EB, and (**c**) ME modes.

Figure 6 shows the variations in the entropy of system output decisions under three different blockchain deployment scenarios (MB, EB, and ME) as the price adjustment speed of channel 1 increases. In general, when the adjustment speed is low, the system remains in a stable state, and the entropy values stay at relatively low levels, indicating limited fluctuations in prices and maintenance efforts as well as strong predictability of the system. As the adjustment speed gradually increases, the system undergoes a transition from stability to bifurcation and eventually to chaos, during which the entropy rises significantly, reflecting the growing complexity and unpredictability of the system. These results suggest that price adjustment speed is a critical factor affecting system stability and complexity, and excessive adjustments may drive both prices and blockchain maintenance efforts into unpredictable chaotic fluctuations.

**Figure 7:**
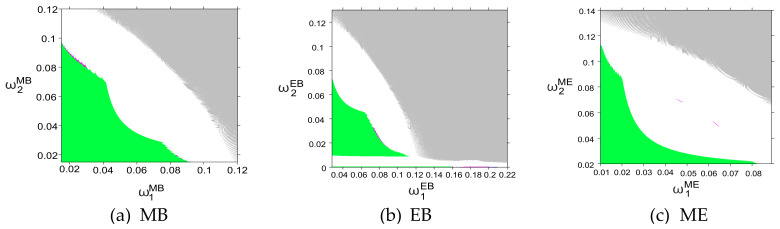
Two-dimensional bifurcation diagrams of the system with respect to the price adjustment speed $\omega_{1}^{\delta }$ of SC1 and $\omega_{2}^{\delta }$ of SC2 in (**a**) MB, (**b**) EB, and (**c**) ME modes.

The above discussion illustrates the impact of the price adjustment speed of a single sales channel on the stability of the system. In order to further explore the impact of the price adjustment speed of both sales channels on the stability of the system, $u_{i}^{\delta }=0.01$ is fixed, and the two-dimensional bifurcation diagram of the system about $\omega_{i}^{\delta }$ in three modes is obtained, as shown in Figure 7. Where green, magenta, white and gray represent the stability, double period bifurcation, chaos and divergence of the system, respectively.

Figure 7 shows that when SC1 and SC2 adjust prices at the same time, the dynamic evolution law of the system also shows the dynamic evolution law of stability, period-doubling bifurcation and chaos. An intuitive comparison shows that in the MB mode, SC1, as a channel for adopting blockchain technology, has a stability threshold of 0.085 for price adjustment speed, while in the EB mode, this threshold is increased to 0.11; similarly, in the EB mode, SC2, as an adopter of blockchain technology, has a stability threshold of 0.07 for price adjustment speed, while in the MB mode, this threshold is increased to 0.09. This is consistent with the conclusion of the figure: blockchain increases the system’s sensitivity to price adjustments, and enterprises need to be more cautious in controlling the adjustment speed to maintain stability.

Overall, from a managerial perspective, these results suggest that CBEC enterprises should strike a balance between transparency and controllability when integrating blockchain into their pricing systems. In practice, platforms such as Alibaba International and Shopee have reported that, although blockchain-based real-time data enhance traceability, they can also amplify short-term price fluctuations during promotional periods. Therefore, firms are advised to adopt gradual, data-driven adjustment mechanisms (e.g., smoothing or bounded learning rates) to maintain dynamic stability. Overall, the findings indicate that blockchain deployment should be coordinated with pricing governance to ensure that improvements in transparency do not compromise system stability.

**Figure 8:**
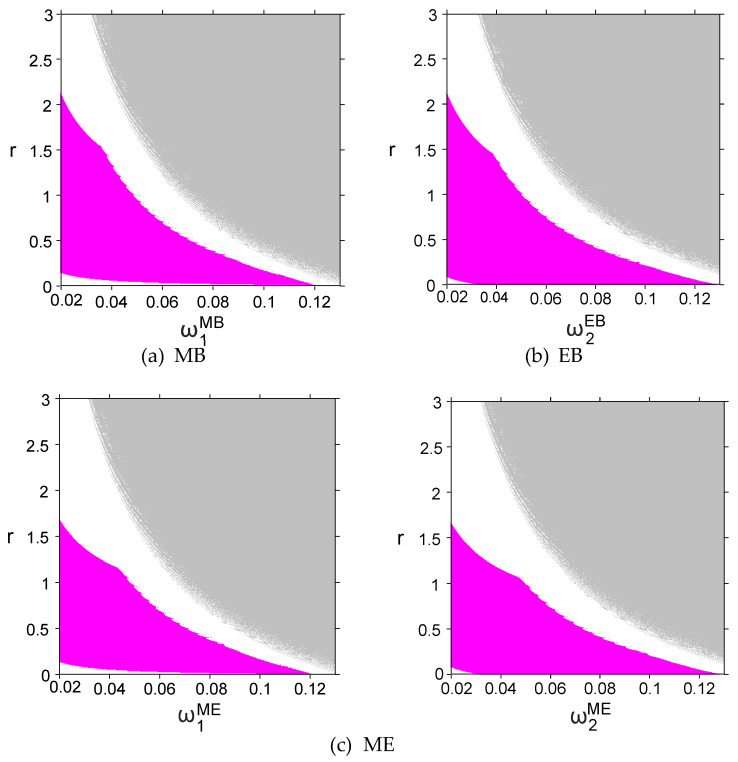
Two-dimensional bifurcation diagrams of the system with respect to tariff rates r and the price adjustment speed $\omega_{i}^{\delta }$ of SCi in (**a**) MB, (**b**) EB, and (**c**) ME modes.

Tariffs, as a key factor affecting product pricing, have a significant impact on the dynamic evolution characteristics of the system. In order to explore the impact of tariff rate r on system stability, Figure 8 shows a two-dimensional bifurcation diagram of system output decisions under four circumstances when r and $\omega_{i}^{\delta }$ increase. Where magenta, white and gray represent the stable, chaotic and divergent states of the system, respectively. Figure 8 shows that, as the tariff rate increases and exceeds a certain threshold, the system goes directly from a stable state to a chaotic state. Specifically, in the MB and EB modes, the stability threshold of the tariff is 2.1, exceeding which the system becomes unstable; while in the ME mode, the threshold drops to 1.6. This shows that in order to maintain the stability of the entire system, importing countries should carefully consider the setting of tariff rates to ensure that they do not exceed the above critical thresholds. In addition, when blockchain technology is deployed simultaneously in dual channels, the system is more sensitive to tariff changes, and the overall stability is further reduced. Enterprises need to pay more attention to the systemic risks brought about by external policy fluctuations in a highly coordinated technical environment.

These findings carry some implications for CBEC enterprises. Firms operating in high-tariff environments need to develop flexible pricing and inventory strategies to anticipate sudden policy-driven market fluctuations. For example, platforms like Alibaba International and Shopee may need to adjust promotional schedules or supplier contracts when tariff changes approach critical thresholds, to prevent systemic instability in supply chain performance. Moreover, when blockchain technology is deployed simultaneously in dual channels, the system is more sensitive to tariff changes, and overall stability is further reduced. This suggests that highly coordinated, technologically advanced supply chains, while improving traceability and transparency, may also amplify the impact of external policy shocks. Therefore, managers must actively monitor policy environments and implement risk mitigation measures—such as dynamic pricing algorithms, tariff hedging strategies, or scenario-based planning—to ensure resilient operation in volatile international markets.

**Figure 9:**
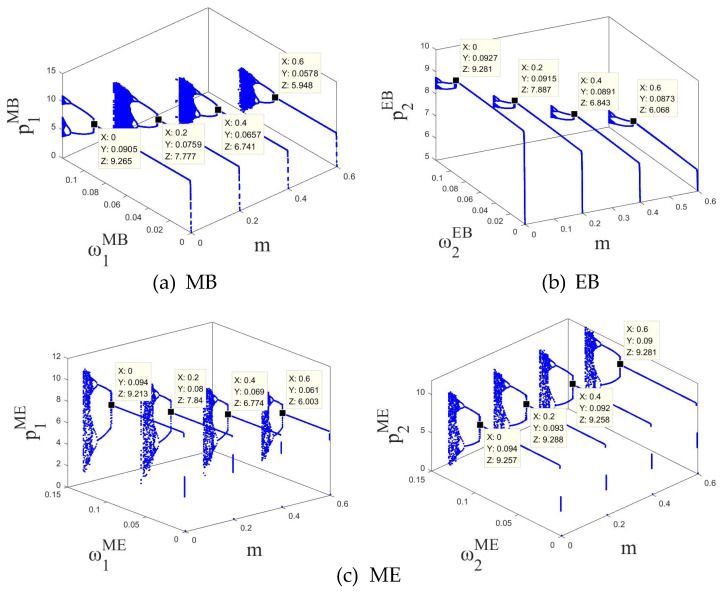
Three-dimensional bifurcation diagrams of the system with respect to commission rate m and the price adjustment speed $\omega_{i}^{\delta }$ of SCi in (**a**) MB, (**b**) EB, and (**c**) ME modes.

Figure 9 shows the impact of the commission rate m of the CBEC platform (E) on system stability in three modes.

As shown in Figure 9, in MB mode, when the commission rate increases from 0% to 60%, the system’s instability bifurcation point drops from $\omega_{1}^{\mathrm{MB}}=0.0905$ to $\omega_{1}^{\mathrm{MB}}=0.0578$, and SC1 enters an unstable state 0.0327 in advance; in EB mode, SC2 becomes unstable 0.0054 in advance; and in ME mode, SC1 and SC2 become unstable 0.0330 and 0.004 in advance, respectively. It can be observed that an increase in the commission rate will generally reduce the stability of the system in all modes. Further comparison shows that SC1 is more sensitive to commissions in the MB mode (0.0327) than SC2 in the EB mode (0.0054), indicating that when the CBEC platform (E) acts as a reseller, the price stability of its channel is more susceptible to changes in commissions. In addition, the sensitivity of SC1 in the ME mode is slightly higher than that of MB (0.0330 > 0.0327), while SC2 is slightly lower than that of EB (0.004 < 0.0054), indicating that the use of blockchain technology in both channels will strengthen the sensitivity of the platform’s resale channel price stability to commissions, while reducing the sensitivity of the direct sales channel. Therefore, excessive commission increases could undermine pricing strategies, leading to unpredictable market responses and potential conflicts with suppliers or resellers. In practice, platforms such as Shopee or Alibaba International could implement adaptive commission mechanisms that dynamically adjust rates based on real-time sales performance, market demand, or channel characteristics. Moreover, the results indicate that integrating blockchain technology across channels amplifies the sensitivity of resale channels to commission changes while stabilizing direct sales channels, implying that technological upgrades should be accompanied by refined financial and pricing governance to mitigate systemic risks. Overall, managers should consider both platform structure and the technological environment when setting commissions to ensure sustainable and stable CBEC operations.

**Figure 10:**
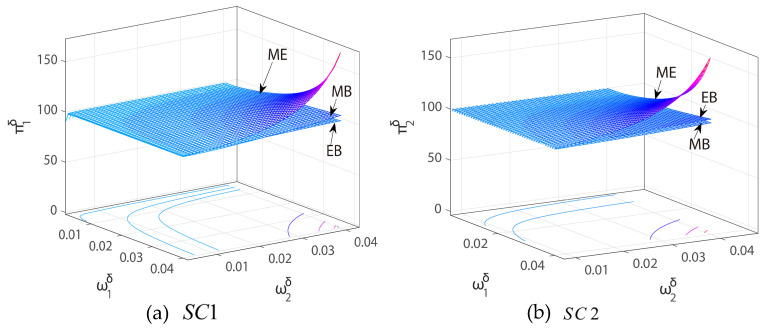
Three-dimensional profit graphs of (**a**) SC1 and (**b**) SC2 with respect to the price adjustment speed $\omega_{1}^{\delta }$ of SC1 and $\omega_{2}^{\delta }$ of SC2.

**Figure 11:**
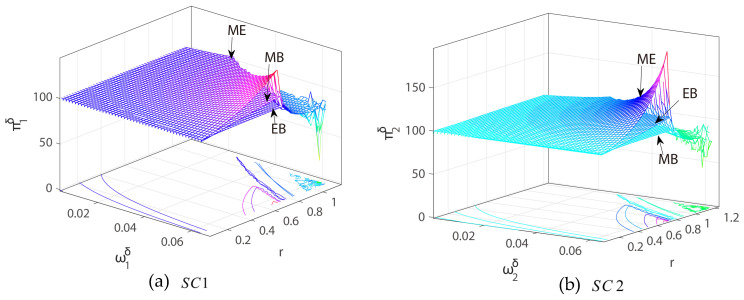
Three-dimensional profit graphs of (**a**) SC1 and (**b**) SC2 with respect to the price adjustment speed $\omega_{i}^{\delta }$ of SCi and tariff rates r.

The above analysis indicates that the price adjustment range and tariff rate are the two core factors that affect the stability of the price competition system, and their changes will directly affect the profit performance of each channel. Figure 10a,b show the profit changes of SC1 and SC2 under different price adjustment speeds, and Figure 11a,b show the profit changes of SC1 and SC2 under their own price adjustment speeds and tariff rates.

As shown in Figure 10, for SC1, in the MB mode (i.e., SC1 adopts blockchain technology), its profit is higher than that in the EB mode (i.e., SC2 adopts blockchain technology); on the contrary, for SC2, the profit in the EB mode is higher than that in the MB mode. This shows that the introduction of blockchain technology can effectively improve the profit level of the channel. In the ME mode (i.e., both SC1 and SC2 adopt blockchain technology), the profits of SC1 and SC2 are higher than the case where only one channel adopts blockchain. This shows that the collaborative adoption of blockchain by two channels can achieve profit synergy gains and enhance the overall performance of the supply chain system. This indicates that the collaborative adoption of blockchain across the two channels can generate profit synergies and enhance the overall performance of the supply chain system, suggesting that CBEC enterprises should consider simultaneous blockchain deployment across multiple channels to maximize efficiency and profitability. In practice, firms such as Alibaba International or Shopee could coordinate blockchain implementation for both direct and reseller channels to ensure traceability, reduce transaction costs, and improve channel coordination, thereby achieving higher combined profits.

In addition, as shown in Figure 11, in the three modes, as the tariff rate continues to rise, the profits of SC1 and SC2 are both on a downward trend and gradually enter a fluctuating state, indicating that excessively high tariffs will weaken channel profitability and increase profit uncertainty. Therefore, when promoting the application of blockchain technology, enterprises should give priority to the collaborative adoption of multiple channels to maximize overall benefits; at the same time, importing countries should not set excessively high tariff rates to avoid compressing the profit space of the supply chain and affecting the profit stability of the supply chain system. This implies that CBEC enterprises must actively monitor international tariff policies and incorporate tariff considerations into pricing and promotional strategies. For example, adjusting pricing speed or promotional intensity in response to anticipated tariff hikes can help maintain profit stability. Moreover, policymakers in importing countries should be aware that setting excessively high tariffs may not only reduce individual channel profitability but also disrupt the coordinated benefits of blockchain-enabled supply chains, suggesting a need for tariff policies that balance revenue goals with the operational stability of cross-border e-commerce.

### 4.2. Dynamic Evolution of System Price Decision-Making When Considering Noise Factors

Since the price adjustment of the supply chain system is often affected by random factors such as exchange rate fluctuations and policy changes, to explore the impact of random disturbances on the dynamic stability of the system, this paper takes $p_{2}^{\mathrm{EB}}$ in EB mode and $p_{1}^{\mathrm{ME}}$ in ME mode as examples, and introduces Gaussian white noise $\rho_{t}$ in their discrete dynamic models to simulate uncontrollable accidental interference factors in the external environment, as shown in formulas (19) and (20). Where the noise variance $\sigma_{p}^{2}$ reflects the intensity of the disturbance and represents the response characteristics of the system under different uncertainty levels.

(19) $$\begin{array}{l} p_{i,t+1}^{\mathrm{EB}}=p_{i,t}^{\mathrm{EB}}+\omega_{i}^{\mathrm{EB}}p_{i,t}^{\mathrm{EB}}\frac{\partial \pi_{i,t}^{\mathrm{EB}}}{\partial p_{i,t}^{\mathrm{EB}}}+\rho_{t},i=1,2\\ b_{2,t+1}^{\mathrm{EB}}=b_{2,t}^{\mathrm{EB}}+\mu_{2}^{\mathrm{EB}}b_{2,t}^{\mathrm{EB}}\frac{\partial \pi_{2,t}^{\mathrm{EB}}}{\partial b_{2,t}^{\mathrm{EB}}}+\rho_{t} \end{array}$$

(20) $$\begin{array}{l} p_{i,t+1}^{\mathrm{ME}}=p_{i,t}^{\mathrm{ME}}+\omega_{i}^{\mathrm{ME}}p_{i,t}^{\mathrm{ME}}\frac{\partial \pi_{i,t}^{\mathrm{ME}}}{\partial p_{i,t}^{\mathrm{ME}}}+\rho_{t},i=1,2\\ b_{i,t+1}^{\mathrm{ME}}=b_{i,t}^{\mathrm{ME}}+\mu_{i}^{\mathrm{ME}}b_{i,t}^{\mathrm{ME}}\frac{\partial \pi_{i,t}^{\mathrm{ME}}}{\partial b_{i,t}^{\mathrm{ME}}}+\rho_{t},i=1,2 \end{array}$$

**Figure 12:**
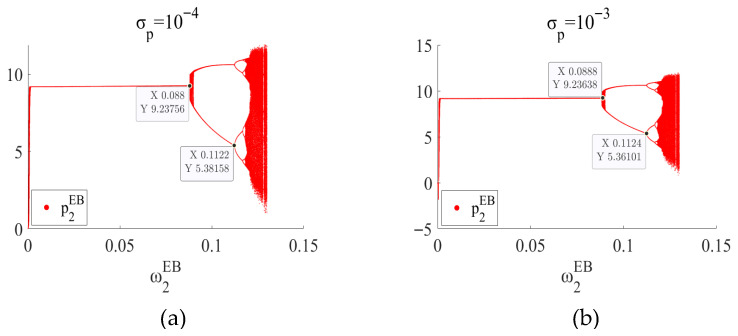
In the EB mode, the bifurcation diagram of price decision $p_{2}^{EB}$ when (**a**) $\sigma_{p}=10^{-4}$ and (**b**) $\sigma_{p}=10^{-3}$.

**Figure 13:**
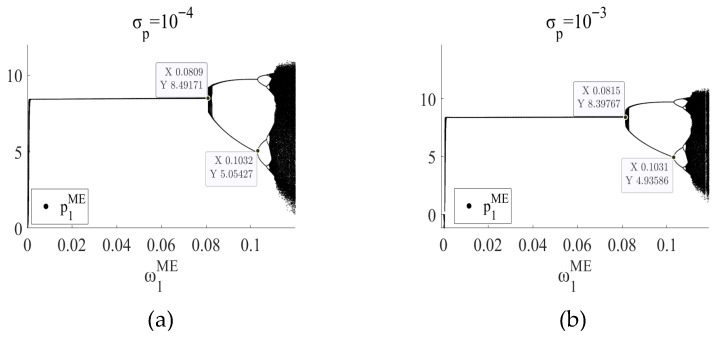
In the ME mode, the bifurcation diagram of price decision $p_{1}^{ME}$ when (**a**) $\sigma_{p}=10^{-4}$ and (**b**) $\sigma_{p}=10^{-3}$.

In the EB mode and ME mode, by setting $\sigma_{p}\in [10^{-4},10^{-3}]$ and after 1000 iterations, the price decision bifurcation diagram under the influence of random disturbance factors is drawn, as shown in Figure 12 and Figure 13, respectively.

As shown in Figure 12, in the EB mode, when $\sigma_{p}$ is $10^{-4}$, the system loses stability at $\omega_{2}^{\mathrm{EB}}=0.0880$ and enters a period-doubling bifurcation state. When $\sigma_{p}$ is $10^{-3}$, this value is $\omega_{2}^{\mathrm{EB}}=0.0888$. In the noiseless case (as shown in the figure), the threshold is $\omega_{2}^{\mathrm{EB}}=0.0878$. As shown in Figure 13, in ME mode, similarly, when $\sigma_{p}$ is $10^{-4}$, the system loses stability at $\omega_{1}^{\mathrm{ME}}=0.0809$. When $\sigma_{p}$ is $10^{-3}$, this value is $\omega_{1}^{\mathrm{ME}}=0.0815$. In the case of no noise (as shown in Figure 5a), the threshold is $\omega_{1}^{\mathrm{ME}}=0.0807$. It can be observed that moderate environmental fluctuations exert a certain “stabilizing” effect, thereby contributing positively to the improvement of system stability. In addition, from the changes in the chaotic region in the figure, it can be observed that moderate noise can also reduce the chaotic region and reduce the uncertainty of the system. However, once the noise exceeds a certain intensity, such as $\sigma_{p}^{2}>10^{-3}$, the system will exceed the iteration region. Therefore, managers should recognize that excessive volatility—such as sudden tariff changes, major supply disruptions, or extreme market shocks—can destabilize the system beyond controllable limits, highlighting the importance of real-time monitoring, risk management, and scenario-based planning to maintain operational stability in CBEC environments.

### 4.3. Chaos Control of the System

When the CBEC system enters a chaotic state, the price decisions of each channel become highly uncertain, and supply chain companies cannot maximize their profits. Therefore, it is necessary to control the chaotic phenomenon of the system’s price decision. This section uses the delayed feedback control method to control the chaotic state of the system under study, that is, when each channel (SC1 and SC2) makes the price decision for the next stage, it fully considers the difference between its current price decision $p_{i,t}^{\delta }$ and the expected output decision $p_{i,t+1}^{\delta }$, and performs feedback control on the price decision for the next stage based on this difference [45].

The control factor k is used to represent the reference degree of SCi to the difference between $p_{i,t}^{\delta }$ and $p_{i,t+1}^{\delta }$. Then, under the delayed feedback control method, the price decision adjustment process of SCi becomes $p_{i,t+1}^{\delta }=p_{i,t}^{\delta }+\omega_{i}^{\delta }p_{i,t}^{\delta }\frac{\partial \pi_{i,t}^{\delta }}{\partial p_{i,t}^{\delta }}+T_{p_{i,t}}^{\delta }$, where $T_{si}^{\sigma }=k(p_{i,t}^{\sigma }-p_{i,t+1}^{\sigma })$. In the case of MB, EB and ME, the delayed feedback control model of the system is shown in expressions (21)–(23).

(21) $$\begin{array}{l} p_{i,t+1}^{\mathrm{MB}}=p_{i,t}^{\mathrm{MB}}+\omega_{i}^{\mathrm{MB}}p_{i,t}^{\mathrm{MB}}\frac{\partial \pi_{i,t}^{\mathrm{MB}}}{\partial p_{i,t}^{MB}}+T_{p_{i,t}}^{\mathrm{MB}},i=1,2\\ b_{1,t+1}^{\mathrm{MB}}=b_{1,t}^{\mathrm{MB}}+\mu_{1}^{\mathrm{MB}}b_{1,t}^{\mathrm{MB}}\frac{\partial \pi_{1,t}^{\mathrm{MB}}}{\partial b_{1,t}^{\mathrm{MB}}}+T_{b_{1,t}}^{\mathrm{MB}} \end{array}$$

(22) $$\begin{array}{l} p_{i,t+1}^{\mathrm{EB}}=p_{i,t}^{\mathrm{EB}}+\omega_{i}^{\mathrm{EB}}p_{i,t}^{\mathrm{EB}}\frac{\partial \pi_{i,t}^{\mathrm{EB}}}{\partial p_{i,t}^{\mathrm{EB}}}+T_{p_{i,t}}^{\mathrm{EB}},i=1,2\\ b_{2,t+1}^{\mathrm{EB}}=b_{2,t}^{\mathrm{EB}}+\mu_{2}^{\mathrm{EB}}b_{2,t}^{\mathrm{EB}}\frac{\partial \pi_{2,t}^{\mathrm{EB}}}{\partial b_{2,t}^{\mathrm{EB}}}+T_{b_{2,t}}^{\mathrm{EB}} \end{array}$$

(23) $$\begin{array}{l} p_{i,t+1}^{\mathrm{ME}}=p_{i,t}^{\mathrm{ME}}+\omega_{i}^{\mathrm{ME}}p_{i,t}^{\mathrm{ME}}\frac{\partial \pi_{i,t}^{\mathrm{ME}}}{\partial p_{i,t}^{\mathrm{ME}}}+T_{p_{i,t}}^{\mathrm{ME}},i=1,2\\ b_{i,t+1}^{\mathrm{ME}}=b_{i,t}^{\mathrm{ME}}+\mu_{i}^{\mathrm{ME}}b_{i,t}^{\mathrm{ME}}\frac{\partial \pi_{i,t}^{\mathrm{ME}}}{\partial b_{i,t}^{\mathrm{ME}}}+T_{b_{i,t}}^{\mathrm{ME}},i=1,2 \end{array}$$

where $T_{p_{i,t}}^{\sigma }=k(p_{i,t}^{\sigma }-p_{i,t+1}^{\sigma })$, $T_{b_{i,t}}^{\sigma }=k(b_{i,t}^{\sigma }-b_{i,t+1}^{\sigma })$.

**Figure 14:**
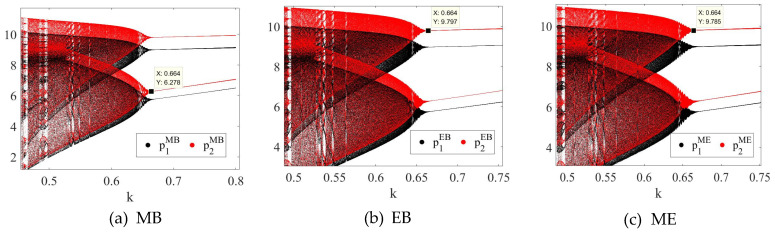
The chaotic control diagrams of the system in the (**a**) MB, (**b**) EB, and (**c**) ME mode.

Figure 14 shows the impact of the control factor k on SC1’s price decision in the three modes. In the three modes, when k>0.664, SC1’s price recovers from a chaotic state to a stable state. This shows that in the three modes, SC1 can effectively control the chaotic state of SC1’s price decision by increasing the reference degree to the difference between $p_{i,t}^{\delta }$ and $p_{i,t+1}^{\delta }$. In addition, since the critical threshold required for the system to recover stability is the same in the three modes, it shows that the sensitivity of different blockchain adoption models to the delayed feedback control method is relatively small. This indicates that CBEC enterprises can employ delayed feedback control strategies as a practical tool to stabilize pricing decisions across different blockchain adoption scenarios. By appropriately tuning the control factor, firms can prevent extreme price fluctuations and maintain more predictable revenue streams, regardless of whether blockchain is adopted by one or both channels. In practice, platforms like Shopee or Alibaba International could integrate such feedback mechanisms into their dynamic pricing algorithms, using real-time monitoring of competitor and reseller prices to adjust their own pricing responses.

## 5. Conclusions

Starting from the perspective of multi-stage dynamic competition, this study investigates, for the first time, the dynamic evolution, entropy, and stability of price competition among channels following the empowerment of CBEC systems by blockchain technology. By constructing discrete dynamic models under three blockchain adoption modes, this study not only theoretically derives the system’s Nash equilibrium solutions and stability conditions in each mode, but also further examines the impact of blockchain technology on the system’s dynamic evolution, the role of noise factors in system stability, and the control of chaotic behavior through numerical simulation analysis. The following summarizes the main findings and contributions of this study:

(1) Blockchain adoption significantly increases the sensitivity of channel price decisions and narrows the tolerance range for initial pricing. Coordinated deployment of blockchain across multiple channels can mitigate asymmetric responses and enhance system coordination. Furthermore, excessive price adjustment speed may drive the system from stability to bifurcation or even chaos, suggesting that firms should carefully regulate the pace of price adjustments following blockchain implementation.
(2) Increases in tariffs and commissions generally destabilize the CBEC system, particularly when platforms act as resellers. Therefore, both enterprises and policymakers should balance tariff and commission settings to maintain supply chain stability. Dual-channel blockchain adoption improves overall profits, but also heightens sensitivity to external shocks, highlighting the need for cautious implementation.
(3) Moderate random disturbances can enhance stability, while excessive noise causes divergence. Finally, delayed feedback control proves effective in restoring system stability from chaotic states across different blockchain deployment modes.

Overall, the findings demonstrate that blockchain improves transparency and coordination, but simultaneously amplifies dynamic sensitivity. Effective pricing control, coordinated adoption, and adaptive policy responses are therefore essential to ensure the long-term stability of blockchain-enabled CBEC systems.

Although this study systematically explores the impact of blockchain technology on the dynamics of CBEC price competition, it has certain limitations. First, the model assumptions are relatively idealized, and the heterogeneity of supply chain members’ behaviors, as well as complex cooperation and competition relationships, are not fully considered. Second, noise is only simulated using Gaussian white noise, and other forms of uncertain disturbances are not addressed. Future research could incorporate more diverse and realistic disturbances, combined with heterogeneous behaviors of supply chain members and multi-level collaboration mechanisms, to enhance the practical applicability of the model.

## Figures and Tables

**Table 1:** Summary of notations.

Symbol	Descriptions
SCi,i=1,2	Sales channel, where1 represents e-commerce platform E as a reseller of P*; 2 represents e-commerce platform E as a retailer of product P*
δ=MB,EB,ME	Blockchain deployment mode, where M means that the blockchain technology is led by overseas manufacturer M and deployed in *SC*1; EB means that e-commerce platform E leads the construction of blockchain technology and deploys blockchain in *SC*2; ME mode means that M and E cooperate to build blockchain technology and introduce blockchain in both *SC*1 and *SC*2
t	Cycle
a	Initial market size of product P*
β	The intensity of competition between the two channels
$p_{i}^{\delta }$	In mode δ , the retail price of product P* in *SCi*
r	Tariff Rates
m	Commission Rate
$b_{i}^{\delta }$	In mode δ , the maintenance efforts of the blockchain on SCi
$g_{i}$	The sensitivity coefficient of the price of SCi’s product P* to the blockchain maintenance effort level
$\eta_{i}^{\delta }$	In mode δ , the maintenance cost coefficient of the blockchain on SCi
$A_{m}$	Fixed construction costs of blockchain
$c_{i}$	SCi’s unit production cost for product P*
$\varepsilon_{1}$	In the ME mode, SCi’s blockchain cost sharing coefficient
$\omega_{i}^{\delta }$	In mode δ, the adjustment speed of SCi’s price decision
$\mu_{i}^{\delta }$	In mode δ, channel i blockchain maintenance effort decision adjustment speed
$\rho_{t}$	Gaussian white noise
$\sigma_{p}^{2}$	Noise variance
k	Control Factors

## Data Availability

Data are contained within the article.

## Appendix A

(A1)
$$\begin{array}{l} p_{1}^{{\mathrm{MB}}^{*}}=-\frac{-2c_{1}{g_{1}}^{2}+2a\eta_{1}+a\beta \eta_{1}+2c_{1}\eta_{1}+\beta c_{2}\eta_{1}}{\left(2{g_{1}}^{2}-4\eta_{1}+\beta^{2}\eta_{1})(1+m+r\right)}\\ p_{2}^{{\mathrm{MB}}^{*}}=-\frac{-a{g_{1}}^{2}-\beta c_{1}{g_{1}}^{2}-c_{2}{g_{1}}^{2}+2a\eta_{1}+a\beta \eta_{1}+\beta c_{1}\eta_{1}+2c_{2}\eta_{1}}{\left(2{g_{1}}^{2}-4\eta_{1}+\beta^{2}\eta_{1})(1+r\right)}\\ b_{1}^{{\mathrm{MB}}^{*}}=-\frac{(2a+a\beta -2c_{1}+\beta^{2}c_{1}+\beta c_{2})g_{1}}{2{g_{1}}^{2}-4\eta_{1}+\beta^{2}\eta_{1}} \end{array}$$

(A2)
$$\begin{array}{l} p_{1}^{{\mathrm{EB}}^{*}}=-\frac{2a\eta_{2}+2c_{1}\eta_{2}+a\beta \eta_{2}+\beta \eta_{2}c_{2}-a{g_{2}}^{2}-c_{1}{g_{2}}^{2}-\beta c_{2}{g_{2}}^{2}}{\left(1+m+r)(-4\eta_{2}+\beta^{2}\eta_{2}+2{g_{2}}^{2}\right)}\\ p_{2}^{{\mathrm{EB}}^{*}}=-\frac{2a\eta_{2}+a\beta \eta_{2}+c_{1}\beta \eta_{2}+2\eta 2c_{2}-2c_{2}{g_{2}}^{2}}{\left(1+r)(-4\eta_{2}+\beta^{2}\eta_{2}+2{g_{2}}^{2}\right)}\\ b_{2}^{{\mathrm{EB}}^{*}}=-\frac{g_{2}(2a+a\beta +c_{1}\beta -2c_{2}+\beta^{2}c_{2})}{-4\eta_{2}+\beta^{2}\eta_{2}+2{g_{2}}^{2}} \end{array}$$

(A3)
$$p_{1}^{{\mathrm{ME}}^{*}}=-\frac{2a\eta_{1}\eta_{2}+2c_{1}\eta_{1}\eta_{2}+a\beta \eta_{1}\eta_{2}+\beta \eta_{1}\eta_{2}c_{2}-2\eta_{2}c_{1}{g_{1}}^{2}-a\eta_{1}{g_{2}}^{2}-c_{1}\eta_{1}{g_{2}}^{2}-\beta \eta_{1}c_{2}{g_{2}}^{2}+c_{1}{g_{1}}^{2}{g_{2}}^{2}}{\left(1+m+r)(-4\eta_{1}\eta_{2}+\beta^{2}\eta_{1}\eta_{2}+2\eta_{2}{g_{1}}^{2}+2\eta_{1}{g_{2}}^{2}-{g_{1}}^{2}{g_{2}}^{2}\right)}$$

(A4)
$$p_{2}^{{\mathrm{ME}}^{*}}=-\frac{2a\eta_{1}\eta_{2}+a\beta \eta_{1}\eta_{2}+c_{1}\beta \eta_{1}\eta_{2}+2\eta_{1}\eta_{2}c_{2}-a\eta_{2}{g_{1}}^{2}-\beta \eta_{2}c_{1}{g_{1}}^{2}-\eta_{2}c_{2}{g_{1}}^{2}-2\eta_{1}c_{2}{g_{2}}^{2}+c_{2}{g_{1}}^{2}{g_{2}}^{2}}{\left(1+r)(-4\eta_{1}\eta_{2}+\beta^{2}\eta_{1}\eta_{2}+2\eta_{2}{g_{1}}^{2}+2\eta_{1}{g_{2}}^{2}-{g_{1}}^{2}{g_{2}}^{2}\right)}$$

(A5)
$$b_{1}^{{\mathrm{ME}}^{*}}=-\frac{g_{1}(2a\eta_{2}+2c_{1}\eta_{2}+a\beta \eta_{2}-4\eta_{2}c_{1}+\beta^{2}\eta_{2}c_{1}+\beta \eta_{2}c_{2}-a{g_{2}}^{2}-c_{1}{g_{2}}^{2}+2c_{1}{g_{2}}^{2}-\beta c_{2}{g_{2}}^{2})}{-4\eta_{1}\eta_{2}+\beta^{2}\eta_{1}\eta_{2}+2\eta_{2}{g_{1}}^{2}+2\eta_{1}{g_{2}}^{2}-{g_{1}}^{2}{g_{2}}^{2}}$$

(A6)
$$b_{2}^{{\mathrm{ME}}^{*}}=-\frac{(2a\eta_{1}+a\beta \eta_{1}+c_{1}\beta \eta_{1}-2\eta_{1}c_{2}+\beta^{2}\eta_{1}c_{2}-a{g_{1}}^{2}-\beta c_{1}{g_{1}}^{2}+c_{2}{g_{1}}^{2})g_{2}}{-4\eta_{1}\eta_{2}+\beta^{2}\eta_{1}\eta_{2}+2\eta_{2}{g_{1}}^{2}+2\eta_{1}{g_{2}}^{2}-{g_{1}}^{2}{g_{2}}^{2}}$$

## Appendix B

(A7)
$$\begin{array}{cc} j_{11}^{\mathrm{MB}}= & 1-2p_{1}^{\mathrm{MB}}{\left(1+m+r\right)}^{2}\omega_{1}^{\mathrm{MB}}+(1+m+r)\omega_{1}^{\mathrm{MB}}(a+c_{1}+b_{1}^{\mathrm{MB}}g_{1}-2p_{1}^{\mathrm{MB}}(1+m+r)+\\ & p_{2}^{\mathrm{MB}}(1+r)\beta )(a+c_{1}+b_{1}^{\mathrm{MB}}g_{1}-2p_{1}^{\mathrm{MB}}(1+m+r)+p_{2}^{\mathrm{MB}}(1+r)\beta ) \end{array}$$

(A8)
$$j_{12}^{\mathrm{MB}}=p_{1}^{\mathrm{MB}}(1+r)(1+m+r)\omega_{1}^{\mathrm{MB}}\beta$$

(A9)
$$j_{13}^{\mathrm{MB}}=g_{1}p_{1}^{\mathrm{MB}}(1+m+r)\omega_{1}^{\mathrm{MB}}$$

(A10)
$$j_{21}^{\mathrm{MB}}=p_{2}^{\mathrm{MB}}(1+r)(1+m+r)\omega_{2}^{\mathrm{MB}}\beta$$

(A11)
$$j_{22}^{\mathrm{MB}}=1-2p_{2}^{MB}{\left(1+r\right)}^{2}\omega_{2}^{\mathrm{MB}}+(1+r)\omega_{2}^{\mathrm{MB}}(a-2p_{2}^{\mathrm{MB}}(1+r)+p_{1}^{\mathrm{MB}}(1+m+r)\beta +c_{2})$$

(A12)
$$j_{23}^{\mathrm{MB}}=0$$

(A13)
$$j_{31}^{\mathrm{MB}}=b_{1}^{\mathrm{MB}}g_{1}(1+m+r)u_{1}^{\mathrm{MB}}$$

(A14)
$$j_{32}^{\mathrm{MB}}=0$$

(A15)
$$j_{33}^{\mathrm{MB}}=1-b_{1}^{\mathrm{MB}}u_{1}^{\mathrm{MB}}\eta_{1}+u_{1}^{\mathrm{MB}}(-b_{1}^{\mathrm{MB}}\eta_{1}+g_{1}(p_{1}^{\mathrm{MB}}(1+m+r)-c_{1}))$$

(A16)
$$j_{11}^{EB}=1-2p_{1}^{EB}{\left(1+m+r\right)}^{2}\omega_{1}^{\mathrm{EB}}+(1+m+r)\omega_{1}^{\mathrm{EB}}(a+c_{1}-2p_{1}^{\mathrm{EB}}(1+m+r)+p_{2}^{\mathrm{EB}}(1+r)\beta )$$

(A17)
$$j_{12}^{\mathrm{EB}}=p_{1}^{\mathrm{EB}}(1+r)(1+m+r)\omega_{1}^{\mathrm{EB}}\beta$$

(A18)
$$j_{13}^{\mathrm{EB}}=0$$

(A19)
$$j_{21}^{\mathrm{EB}}=p_{2}^{\mathrm{EB}}(1+r)(1+m+r)\omega_{2}^{\mathrm{EB}}\beta$$

(A20)
$$j_{22}^{\mathrm{EB}}=1-2p_{2}^{\mathrm{EB}}{\left(1+r\right)}^{2}\omega_{2}^{\mathrm{EB}}+(1+r)\omega_{2}^{\mathrm{EB}}(a-2p_{2}^{\mathrm{EB}}(1+r)+p_{1}^{\mathrm{EB}}(1+m+r)\beta +c_{2}+b_{2}^{\mathrm{EB}}g_{2})$$

(A21)
$$j_{23}^{\mathrm{EB}}=p_{2}^{\mathrm{EB}}(1+r)\omega_{2}^{\mathrm{EB}}g_{2}$$

(A22)
$$j_{31}^{\mathrm{EB}}=0$$

(A23)
$$j_{32}^{\mathrm{EB}}=b_{1}^{\mathrm{EB}}g_{2}(1+r)u_{1}^{\mathrm{EB}}$$

(A24)
$$j_{33}^{\mathrm{EB}}=-b_{1}^{\mathrm{EB}}u_{1}^{\mathrm{EB}}\eta_{2}$$

## Appendix C

(A25)
$$\begin{array}{cc} j_{11}^{\mathrm{ME}}= & 1-2p_{1}^{\mathrm{ME}}{\left(1+m+r\right)}^{2}\omega_{1}^{\mathrm{ME}}+(1+m+r)\omega_{1}^{\mathrm{ME}}(a+c_{1}-2p_{1}^{\mathrm{ME}}(1+m+r)+\\ & p_{2}^{\mathrm{ME}}(1+r)\beta +b_{1}^{\mathrm{ME}}g_{1}) \end{array}$$

(A26)
$$j_{12}^{\mathrm{ME}}=p_{1}^{\mathrm{ME}}(1+r)(1+m+r)\omega_{1}^{\mathrm{ME}}\beta$$

(A27)
$$j_{13}^{\mathrm{ME}}=p_{1}^{\mathrm{ME}}(1+m+r)\omega_{1}^{\mathrm{ME}}g_{1}$$

(A28)
$$j_{14}^{\mathrm{ME}}=0$$

(A29)
$$j_{21}^{\mathrm{ME}}=p_{2}^{\mathrm{ME}}(1+r)(1+m+r)\omega_{2}^{\mathrm{ME}}\beta$$

(A30)
$$j_{22}^{\mathrm{ME}}=1-2p_{2}^{\mathrm{ME}}{\left(1+r\right)}^{2}\omega_{2}^{\mathrm{ME}}+(1+r)\omega_{2}^{\mathrm{ME}}(a-2p_{2}^{\mathrm{ME}}(1+r)+p_{1}^{\mathrm{ME}}(1+m+r)\beta +c_{2}+b_{2}^{\mathrm{ME}}g_{2})$$

(A31)
$$j_{23}^{\mathrm{ME}}=0$$

(A32)
$$j_{24}^{\mathrm{ME}}=p_{2}^{\mathrm{ME}}(1+r)\omega_{2}^{\mathrm{ME}}g_{2}$$

(A33)
$$j_{31}^{\mathrm{ME}}=b_{1}^{\mathrm{ME}}(1+m+r)u_{1}^{\mathrm{ME}}g_{1}$$

(A34)
$$j_{32}^{\mathrm{ME}}=0$$

(A35)
$$j_{33}^{\mathrm{ME}}=1-b_{1}^{\mathrm{ME}}u_{1}^{\mathrm{ME}}\eta_{1}+u_{1}^{\mathrm{ME}}(-b_{1}^{\mathrm{ME}}\eta_{1}+(p_{1}^{\mathrm{ME}}(1+m+r)-c_{1})g_{1})$$

(A36)
$$j_{34}^{\mathrm{ME}}=0$$

(A37)
$$j_{41}^{\mathrm{ME}}=0$$

(A38)
$$j_{42}^{\mathrm{ME}}=b_{2}^{\mathrm{ME}}(1+r)u_{2}^{\mathrm{ME}}g_{2}$$

(A39)
$$j_{43}^{\mathrm{ME}}=0$$

(A40)
$$j_{44}^{\mathrm{ME}}=1-b_{2}^{\mathrm{ME}}u_{2}^{\mathrm{ME}}\eta_{2}+u_{2}^{\mathrm{ME}}(-b_{2}^{\mathrm{ME}}\eta_{2}+(p_{2}^{\mathrm{ME}}(1+r)-c_{2})g_{2})$$

## Appendix D

(A41)
$$B_{1}^{\mathrm{ME}}=-j_{11}^{\mathrm{ME}}-j_{22}^{\mathrm{ME}}-j_{33}^{\mathrm{ME}}-j_{44}^{\mathrm{ME}}$$

(A42)
$$\begin{array}{cc} B_{2}^{\mathrm{ME}}= & -j_{12}^{\mathrm{ME}}j_{21}^{\mathrm{ME}}+j_{11}^{\mathrm{ME}}j_{22}^{\mathrm{ME}}-j_{13}^{\mathrm{ME}}j_{31}^{\mathrm{ME}}-j_{23}^{\mathrm{ME}}j_{32}^{\mathrm{ME}}+j_{11}^{\mathrm{ME}}j_{33}^{\mathrm{ME}}+j_{22}^{\mathrm{ME}}j_{33}^{\mathrm{ME}}-\\ & j_{14}^{\mathrm{ME}}j_{41}^{\mathrm{ME}}-j_{24}^{\mathrm{ME}}j_{42}^{\mathrm{ME}}-j_{34}^{\mathrm{ME}}j_{43}^{\mathrm{ME}}+j_{11}^{\mathrm{ME}}j_{44}^{\mathrm{ME}}+j_{22}^{\mathrm{ME}}j_{44}^{\mathrm{ME}}+j_{33}^{\mathrm{ME}}j_{44}^{\mathrm{ME}} \end{array}$$

(A43)
$$\begin{array}{cc} B_{3}^{\mathrm{ME}}= & j_{13}^{\mathrm{ME}}j_{22}^{\mathrm{ME}}j_{31}^{\mathrm{ME}}-j_{12}^{\mathrm{ME}}j_{23}^{\mathrm{ME}}j_{31}^{\mathrm{ME}}-j_{13}^{\mathrm{ME}}j_{21}^{\mathrm{ME}}j_{32}^{\mathrm{ME}}+j_{11}^{\mathrm{ME}}j_{23}^{\mathrm{ME}}j_{32}^{\mathrm{ME}}+j_{12}^{\mathrm{ME}}j_{21}^{\mathrm{ME}}j_{33}^{\mathrm{ME}}-\\ & j_{11}^{\mathrm{ME}}j_{22}^{\mathrm{ME}}j_{33}^{\mathrm{ME}}+j_{14}^{\mathrm{ME}}j_{22}^{\mathrm{ME}}j_{41}^{\mathrm{ME}}-j_{12}^{\mathrm{ME}}j_{24}^{\mathrm{ME}}j_{41}^{\mathrm{ME}}+j_{14}^{\mathrm{ME}}j_{33}^{\mathrm{ME}}j_{41}^{\mathrm{ME}}-j_{13}^{\mathrm{ME}}j_{34}^{\mathrm{ME}}j_{41}^{\mathrm{ME}}-j_{14}^{\mathrm{ME}}j_{21}^{\mathrm{ME}}j_{42}^{\mathrm{ME}}+\\ & j_{11}^{\mathrm{ME}}j_{24}^{\mathrm{ME}}j_{42}^{\mathrm{ME}}+j_{24}^{\mathrm{ME}}j_{33}^{\mathrm{ME}}j_{42}^{\mathrm{ME}}-j_{23}^{\mathrm{ME}}j_{34}^{\mathrm{ME}}j_{42}^{\mathrm{ME}}-j_{14}^{\mathrm{ME}}j_{31}^{\mathrm{ME}}j_{43}^{\mathrm{ME}}-j_{24}^{\mathrm{ME}}j_{32}^{\mathrm{ME}}j_{43}^{\mathrm{ME}}+j_{11}^{\mathrm{ME}}j_{34}^{\mathrm{ME}}j_{43}^{\mathrm{ME}}+\\ & j_{22}^{\mathrm{ME}}j_{34}^{\mathrm{ME}}j_{43}^{\mathrm{ME}}+j_{12}^{\mathrm{ME}}j_{21}^{\mathrm{ME}}j_{44}^{\mathrm{ME}}-j_{11}^{\mathrm{ME}}j_{22}^{\mathrm{ME}}j_{44}^{\mathrm{ME}}+j_{13}^{\mathrm{ME}}j_{31}^{\mathrm{ME}}j_{44}^{\mathrm{ME}}+j_{23}^{\mathrm{ME}}j_{32}^{\mathrm{ME}}j_{44}^{\mathrm{ME}}-j_{11}^{\mathrm{ME}}j_{33}^{\mathrm{ME}}j_{44}^{\mathrm{ME}}-\\ & j_{22}^{\mathrm{ME}}j_{33}^{\mathrm{ME}}j_{44}^{\mathrm{ME}} \end{array}$$

(A44)
$$\begin{array}{cc} B_{4}^{\mathrm{ME}}= & j_{14}^{\mathrm{ME}}j_{23}^{\mathrm{ME}}j_{32}^{\mathrm{ME}}j_{41}^{\mathrm{ME}}-j_{13}^{\mathrm{ME}}j_{24}^{\mathrm{ME}}j_{32}^{\mathrm{ME}}j_{41}^{\mathrm{ME}}-j_{14}^{\mathrm{ME}}j_{22}^{\mathrm{ME}}j_{33}^{\mathrm{ME}}j_{41}^{\mathrm{ME}}+j_{12}^{\mathrm{ME}}j_{24}^{\mathrm{ME}}j_{33}^{\mathrm{ME}}j_{41}^{\mathrm{ME}}+j_{13}^{\mathrm{ME}}j_{22}^{\mathrm{ME}}j_{34}^{\mathrm{ME}}j_{41}^{\mathrm{ME}}-\\ & j_{12}^{\mathrm{ME}}j_{23}^{\mathrm{ME}}j_{34}^{\mathrm{ME}}j_{41}^{\mathrm{ME}}-j_{14}^{\mathrm{ME}}j_{23}^{\mathrm{ME}}j_{31}^{\mathrm{ME}}j_{42}^{\mathrm{ME}}+j_{13}^{\mathrm{ME}}j_{24}^{\mathrm{ME}}j_{31}^{\mathrm{ME}}j_{42}^{\mathrm{ME}}+j_{14}^{\mathrm{ME}}j_{21}^{\mathrm{ME}}j_{33}^{\mathrm{ME}}j_{42}^{\mathrm{ME}}-j_{11}^{\mathrm{ME}}j_{24}^{\mathrm{ME}}j_{33}^{\mathrm{ME}}j_{42}^{\mathrm{ME}}-\\ & j_{13}^{\mathrm{ME}}j_{21}^{\mathrm{ME}}j_{34}^{\mathrm{ME}}j_{42}^{\mathrm{ME}}+j_{11}^{\mathrm{ME}}j_{23}^{\mathrm{ME}}j_{34}^{\mathrm{ME}}j_{42}^{\mathrm{ME}}+j_{14}^{\mathrm{ME}}j_{22}^{\mathrm{ME}}j_{31}^{\mathrm{ME}}j_{43}^{\mathrm{ME}}-j_{12}^{\mathrm{ME}}j_{24}^{\mathrm{ME}}j_{31}^{\mathrm{ME}}j_{43}^{\mathrm{ME}}-j_{14}^{\mathrm{ME}}j_{21}^{\mathrm{ME}}j_{32}^{\mathrm{ME}}j_{43}^{\mathrm{ME}}+\\ & j_{11}^{\mathrm{ME}}j_{24}^{\mathrm{ME}}j_{32}^{\mathrm{ME}}j_{43}^{\mathrm{ME}}+j_{12}^{\mathrm{ME}}j_{21}^{\mathrm{ME}}j_{34}^{\mathrm{ME}}j_{43}^{\mathrm{ME}}-j_{11}^{\mathrm{ME}}j_{22}^{\mathrm{ME}}j_{34}^{\mathrm{ME}}j_{43}^{\mathrm{ME}}-j_{13}^{\mathrm{ME}}j_{22}^{\mathrm{ME}}j_{31}^{\mathrm{ME}}j_{44}^{\mathrm{ME}}+j_{12}^{\mathrm{ME}}j_{23}^{\mathrm{ME}}j_{31}^{\mathrm{ME}}j_{44}^{\mathrm{ME}}+\\ & j_{13}^{\mathrm{ME}}j_{21}^{\mathrm{ME}}j_{32}^{\mathrm{ME}}j_{44}^{\mathrm{ME}}-j_{11}^{\mathrm{ME}}j_{23}^{\mathrm{ME}}j_{32}^{\mathrm{ME}}j_{44}^{\mathrm{ME}}-j_{12}^{\mathrm{ME}}j_{21}^{\mathrm{ME}}j_{33}^{\mathrm{ME}}j_{44}^{\mathrm{ME}}+j_{11}^{\mathrm{ME}}j_{22}^{\mathrm{ME}}j_{33}^{\mathrm{ME}}j_{44}^{\mathrm{ME}} \end{array}$$
